# Optimizing DNA Extraction Methods for Nanopore Sequencing of Neisseria gonorrhoeae Directly from Urine Samples

**DOI:** 10.1128/JCM.01822-19

**Published:** 2020-02-24

**Authors:** Teresa L. Street, Leanne Barker, Nicholas D. Sanderson, James Kavanagh, Sarah Hoosdally, Kevin Cole, Robert Newnham, Mathyruban Selvaratnam, Monique Andersson, Martin J. Llewelyn, Justin O’Grady, Derrick W. Crook, David W. Eyre

**Affiliations:** a Nuffield Department of Clinical Medicine, University of Oxford, John Radcliffe Hospital, Oxford, United Kingdom; b Department of Microbiology and Infection, Royal Sussex County Hospital, Brighton, United Kingdom; c Microbiology Laboratory, John Radcliffe Hospital, Oxford University Hospitals NHS Foundation Trust, Oxford, United Kingdom; d Quadram Institute Bioscience, Norwich, United Kingdom; e National Institute for Health Research Oxford Biomedical Research Centre, John Radcliffe Hospital, Oxford, United Kingdom; f Big Data Institute, University of Oxford, Oxford, United Kingdom; University of Iowa College of Medicine

**Keywords:** DNA extraction, Nanopore sequencing, *Neisseria gonorrhoeae*, whole-genome sequencing

## Abstract

Empirical gonorrhea treatment at initial diagnosis reduces onward transmission. However, increasing resistance to multiple antibiotics may necessitate waiting for culture-based diagnostics to select an effective treatment. There is a need for same-day culture-free diagnostics that identify infection and detect antimicrobial resistance. We investigated if Nanopore sequencing can detect sufficient Neisseria gonorrhoeae DNA to reconstruct whole genomes directly from urine samples. We used N. gonorrhoeae-spiked urine samples and samples from gonorrhea infections to determine optimal DNA extraction methods that maximize the amount of N. gonorrhoeae DNA sequenced while minimizing contaminating host DNA. In simulated infections, the Qiagen UCP pathogen mini kit provided the highest ratio of N. gonorrhoeae to human DNA and the most consistent results. Depletion of human DNA with saponin increased N. gonorrhoeae yields in simulated infections but decreased yields in clinical samples. In 10 urine samples from men with symptomatic urethral gonorrhea, ≥92.8% coverage of an N. gonorrhoeae reference genome was achieved in all samples, with ≥93.8% coverage breath at ≥10-fold depth in 7 (70%) samples. In simulated infections, if ≥10^4^ CFU/ml of N. gonorrhoeae was present, sequencing of the large majority of the genome was frequently achieved. N. gonorrhoeae could also be detected from urine in cobas PCR medium tubes and from urethral swabs and in the presence of simulated *Chlamydia* coinfection. Using Nanopore sequencing of urine samples from men with urethral gonorrhea, sufficient data can be obtained to reconstruct whole genomes in the majority of samples without the need for culture.

## Abstract (precis)

Empirical gonorrhea treatment at initial diagnosis reduces onward transmission. However, increasing resistance to multiple antibiotics may necessitate waiting for culture-based diagnostics to select an effective treatment. There is a need for same-day culture-free diagnostics that identify infection and detect antimicrobial resistance. We investigated if Nanopore sequencing can detect sufficient Neisseria gonorrhoeae DNA to reconstruct whole genomes directly from urine samples.

## INTRODUCTION

Multidrug-resistant Neisseria gonorrhoeae infection is a substantial public health threat (1, 2). To reduce the spread of antimicrobial resistance, empirical dual therapy with single-dose azithromycin and ceftriaxone, the last two mainstream treatment options, is widely recommended (2). However, increasing azithromycin resistance potentially undermines this approach, leaving ceftriaxone empirical monotherapy as a last option (3). However, ceftriaxone-resistant cases have been recently reported in several countries worldwide (4, 5).

Single-dose gonorrhea treatment at the point of initial diagnosis reduces onward transmission (6). However, rising resistance rates may necessitate delays of up to several days while culture-based susceptibility testing is performed to direct effective treatment, potentially increasing transmission of resistant organisms. Therefore, there is a need for same-day culture-free diagnostics that are able to confirm both the presence of infection and detect antimicrobial resistance (7).

Multiple PCR-based assays for resistance markers have been developed, e.g., references 8 and 9; however, the diversity of resistance mutations and variants within N. gonorrhoeae make developing comprehensive panels challenging (7). In contrast, clinical metagenomics has the potential to detect N. gonorrhoeae and any antimicrobial resistance determinants present via direct sequencing of all DNA present in a clinical sample (10). Previous studies have shown this is possible for N. gonorrhoeae using Illumina sequencing (11), but this approach is not suitable for rapid near-patient deployment. Furthermore, current approaches are limited by obtaining sufficient pathogen DNA against a background of human host DNA and DNA from other bacteria (12). Several approaches have been used to increase pathogen DNA yields, e.g., microbial enrichment via immunomagnetic separation (13) for sequencing Chlamydia trachomatis from urine or capture of pathogen DNA after DNA extraction using RNA baits (14). Several commercial kits exist for selective depletion of human DNA (12). Despite this, obtaining sufficient pathogen DNA for whole-genome reconstruction from metagenomic sequencing remains challenging, and the short reads generated by Illumina sequencing make it difficult to accurately assign resistance determinants to a specific species in metagenomic samples.

The Oxford Nanopore sequencing platform has the potential to overcome these challenges and deliver N. gonorrhoeae and antimicrobial resistance detection in a benchtop format that yields results within a few hours. The long reads generated have the potential to link antimicrobial resistance determinants accurately to a given species, as a greater genetic context is provided. Here, we build on previous work applying Nanopore sequencing for the diagnosis of urinary tract infection (15) and develop optimized laboratory protocols for sequencing N. gonorrhoeae directly from patient urine samples.

## MATERIALS AND METHODS

### Samples.

Urine samples collected as part of routine clinical testing of patients at sexual health clinics in Oxfordshire, UK, were obtained from the Microbiology Laboratory of Oxford University Hospitals NHS Foundation Trust (OUH) and were used for initial method development. Samples were tested for N. gonorrhoeae and Chlamydia trachomatis using the BD Viper system, with confirmatory testing for N. gonorrhoeae undertaken using the BD Max platform (Becton, Dickinson, Wokingham, Berkshire, UK). Samples that would otherwise have been discarded were obtained for research use after completion of routine testing.

Additionally, samples were collected from participants recruited at sexual health clinics at the Churchill Hospital, OUH, and Brighton and Sussex University Hospitals, NHS Trust, Brighton, UK. Male patients presenting with symptomatic urethritis were eligible to participate and were recruited following informed consent. In this study, urine samples from participants recruited in Brighton were used to test the performance of metagenomic sequencing in clinical infection. Urine samples were collected into universal tubes containing boric acid (Medline Scientific, Chalgrove, Oxfordshire, UK) and then placed into cobas PCR medium tubes (Roche Molecular Systems, Pleasanton, CA, USA) for stabilization during transportation to Oxford. Urethral swabs were collected and placed into Sigma VCM preservation medium (MWE, Corsham, Wiltshire, UK). This study was conducted with NHS Research Ethics approval (reference 19/EM/0029).

### Human DNA depletion and microbial DNA extraction.

To optimize laboratory methods for the selective extraction of high-quality, long-fragment N. gonorrhoeae DNA directly from urine, we tested 16 approaches as follows: four DNA extraction methods in combination with either no human cell/DNA depletion or one of three human cell/DNA depletion protocols (see Fig. S1 in the supplemental material). For each approach, N. gonorrhoeae nucleic acid amplification test (NAAT)-negative urine samples were spiked at 10^3^, 10^5^, and 10^7^ CFU/ml with a N. gonorrhoeae reference strain. Testing at each dilution was undertaken in triplicate using three N. gonorrhoeae reference strains, namely, WHO F, WHO V, and WHO X (obtained from Public Health England’s National Collection of Type Cultures), and a starting urine volume of 3 ml. Multiple negative urine samples were pooled to allow the same baseline urine to be tested across each depletion and extraction protocol. The number of CFU/ml present in each spike was initially determined using dilutions of a 0.5 McFarland standard; the resulting dilutions were cultured at 37°C with 5% CO_2_ for 24 h on lysed GC selective Agar (Oxoid, Basingstoke, Hampshire, UK) to enable the final N. gonorrhoeae CFU/ml achieved to be measured.

The following three human cell/DNA depletion methods were tested: differential centrifugation, saponin-based differential lysis followed by nuclease digestion, and the MolYsis Basic5 kit (Molzym, Bremen, Germany). Urine samples were also processed with no human DNA depletion, as a negative control. Differential centrifugation was performed as previously described; it relies on separating human cells based on their larger size/mass with an slower centrifugation step, before applying a more rapid centrifugation to the initial supernatant to obtain the bacterial cells (15). The bacterial cell pellet was washed with 1 ml phosphate-buffered saline (PBS) before proceeding to DNA extraction. Saponin-based differential lysis and nuclease digestion were performed as previously described (16). Saponin acts as a detergent, with reported greater activity against human cells, which are lysed, and the released DNA is then digested prior to extraction of DNA from the remaining intact bacterial cells. Human DNA depletion and enrichment of microbial DNA with the MolYsis Basic5 kit, a proprietary kit, was performed per the manufacturer’s instructions.

The four DNA extraction methods assessed included mechanical lysis followed by ethanol precipitation, as described previously (17); the MagMAX total nucleic acid isolation kit (ThermoFisher Scientific, Waltham, MA, USA); QIAamp UCP pathogen mini kit (Qiagen, Hilden, Germany); and i-genomic urine DNA extraction mini kit (iNtRON Bio, Burlington, MA, USA); which were all performed following the manufacturer’s instructions. Extracted DNA was purified using AMPure XP solid-phase reversible immobilization (SPRI) beads (Beckman Coulter, High Wycombe, UK), eluted in 26 μl of Tris-EDTA (TE) buffer, and quantified using a Qubit 2.0 fluorometer (Life Technologies, Paisley, UK).

### PCR analysis of N. gonorrhoeae and human DNA.

Quantitative real-time PCR (qPCR) was performed to determine the relative amounts of both N. gonorrhoeae and human DNA in the DNA extracts from the initial laboratory optimization methods. qPCR was performed on a Stratagene MX3005P qPCR system (Agilent Technologies, Santa Clara, CA, USA) using Luna universal probe qPCR master mix (New England BioLabs, Ipswich, MA, USA). Primers and probes were used to target the β-actin gene for human DNA detection (18) and the *porA* pseudogene for detection of N. gonorrhoeae (papTM) (19). Reactions were performed in 20 μl, with 2 μl of template DNA, 0.4 μM each primer, and 0.2 μM of the probe. Cycling conditions were an initial denaturation at 95°C for 1 min, followed by 40 cycles of 95°C denaturation for 15 s, and 60°C extension for 30 s. N. gonorrhoeae genomic DNA, extracted from cultures of WHO F, WHO V, and WHO X reference strains, was diluted to 100,000 genome copies per μl then serially diluted to 10 genome copies per μl, and used to create copy number standard curves. Human genomic DNA (Promega, Madison, WI, USA) was diluted to 10,000 genome copies per μl then serially diluted to 10 genome copies per μl and used to create a human DNA copy number standard curve. Negative controls, replacing template DNA with water, were also performed. All qPCR assays were performed in triplicate, and the mean value was used in analyses.

### In-depth spiking and metagenomic whole-genome sequencing.

Using the optimal laboratory method determined in the spiking experiments, an extended set of spiking experiments were performed to determine the limit of detection of this protocol for N. gonorrhoeae DNA. A total 3 ml of N. gonorrhoeae NAAT-negative pooled urine, was spiked with one of the WHO F, WHO V, or WHO X N. gonorrhoeae reference strains, using dilutions of a 0.5 McFarland standard, to target 10^2^, 10^3^, 10^4^, 10^5^, 10^6,^ or 10^7^ CFU/ml, using the three reference strains as replicates (*n* = 19, including an unspiked urine sample as a negative control). DNA extracts were assessed for human and microbial DNA content by metagenomic sequencing. Libraries were prepared for sequencing on an GridION instrument (Oxford Nanopore Technologies [ONT], Oxford, UK) using the Rapid PCR barcoding kit (catalog number SQK-RPB004) (ONT), with modifications to the manufacturers’ protocol as described by Charalampous et al. (16) Briefly, up to 10 ng of input DNA and 2.5 μl of fragmentation mix (FRM) were used in the tagmentation reaction with a final volume of 10 μl. Tagmentation describes transposase-based DNA fragmentation and tagging with adapters prior to library preparation. For samples that were not able to achieve 10 ng, a total volume of 7.5 μl was used in the tagmentation reaction regardless of the quantity of DNA this represented. The PCR was performed in a double volume of 100 μl, with 25 cycles and an elongation time of 6 minutes. After the PCR, DNA was purified with AMPure XP SPRI beads (Beckman Coulter, High Wycombe, UK) and eluted in 10 μl. Initially, all samples were sequenced individually on ONT FLO-MIN106D (v.R9.4.1) flow cells regardless of the quantity of DNA after PCR. Subsequently, only samples with amounts of DNA quantifiable by a Qubit fluorometer after PCR were used for sequencing. Between 3 and 85 fmol of library was loaded per flow cell. One sample was run per flow cell for all experiments.

### DNA extraction in simulated coinfection.

To assess sequencing in the presence of coinfection, chlamydia NAAT-positive, N. gonorrhoeae NAAT-negative urine collected from three patients was used. Totals of 10^2^ and 10^4^ CFU/ml of one of three N. gonorrhoeae reference strains (WHO F, WHO X, and WHO V) were individually spiked into 3 ml of each urine sample. An unspiked sample from each patient was used as a negative control (6 spiked samples and 3 control samples in total). Following spiking, each sample was split and DNA was extracted with either the optimal extraction protocol of saponin-based differential lysis and the QIAamp kit or with saponin-based differential lysis followed by mechanical lysis and ethanol precipitation (to ascertain whether it was possible to recover N. gonorrhoeae and C. trachomatis DNA and to assess any potential bias in DNA recovery between the two methods; *n* = 18 overall). DNA was sequenced on an ONT GridION instrument as described above.

### DNA extraction from NAAT-positive urine samples and urethral swabs.

Samples from study participants were used to assess the real-world performance of our methods in N. gonorrhoeae NAAT-positive urine samples from men with urethral gonorrhea infection. Clinical samples were tested with the FTD gonorrhea confirmation NAAT assay (Fast Track Diagnostics, Sliema, Malta). DNA was extracted from 3 ml of urine from 10 individual participants using the QIAamp UCP pathogen mini kit (chosen on the basis of the simulated infection experiments) with and without saponin-based human DNA depletion. DNA was sequenced on an ONT GridION instrument as described above. A single flow cell was used for each urine sample, with DNA extracts from the same sample with and without saponin treatment multiplexed on each flow cell. Urine from 4 of the 10 individual participants was also collected into cobas PCR medium tubes designed for diagnostic molecular N. gonorrhoeae and C. trachomatis testing. DNA was extracted from 4 ml obtained from these tubes using the QIAamp kit (without saponin treatment), both with or without a prior mechanical lysis step using bead beating as previously described (17), with the resulting two DNA extracts from each sample multiplexed as a pair on a single flow cell. Urethral swabs were obtained from nine of the participants and stored in Sigma VCM preservation medium for transport. DNA was extracted from 3 ml of this medium, vortexed for 30 seconds using the QIAamp UCP pathogen mini kit without saponin treatment, and sequenced as described above with a single sample per flow cell.

### Bioinformatic analysis.

Nanopore sequences were base called using Guppy (v.1.8.5; ONT) and demultiplexed with Porechop (v.0.2.4) (https://github.com/rrwick/Porechop) using the default settings. Reads were taxonomically classified using Centrifuge (b.1.0.4-beta) (20), with “–min-hitlen 16 -k 1 –mm” settings, using a database of NCBI RefSeq bacterial and viral genomes submitted by August 10, 2018 and the human reference genome (GRCh38). Reads classified as human were securely deleted prior to subsequent analyses. Improvements in demultiplexing technology occurred during the study. To reduce the number of reads not successfully demultiplexed, FAST5 files containing sequence data including barcodes, from samples obtained from participants, were base called and demultiplexed again using an updated version of Guppy (3.3.0+ef22818; ONT). As the large majority of human reads had already been removed from the FAST5 files following initial demultiplexing with porechop, results presenting numbers of human reads and total reads are presented including data demultiplexed by porechop and guppy; all other analyses are based on guppy data alone. Base calling was done with the “high accuracy” (HAC) model and recommended kit and flow cell configurations. Barcode demultiplexing was done with default parameters.

Classified reads were aligned to reference genomes using Minimap2 (v.2.17-r954-dirty) (21) with “-L -ax map-ont” settings and filtered to a mapQ of 50 by SAMtools (v.1.9-211-gef8e24f using htslib v.1.9-446-gc673947) (22) using “samtools view” as described in the CRuMPIT workflow (23) (https://gitlab.com/ModernisingMedicalMicrobiology/CRuMPIT) (commit 33deb08b). CRuMPIT was used to generate per species metrics for read numbers, bases, and coverage depth and breadth. Read coverage depth is the number of reads that align to a given base in the reference genome and is typically reported as the mean across the whole genome, e.g., 20-fold or 20× coverage. Coverage breadth is the percentage of the reference genome covered by at least one read but can also be reported as the percentage of the reference genome covered by at least a higher number of reads, e.g., 10. Within CRuMPIT, the depth and breadth of the mapped reads were assessed using SAMtools with the “samtools depth” command (22). Additional plots were generated using the following python libraries: pandas (v.0.25.1) (24), seaborn (v.0.9.0), and matplotlib (v.2.2.4) (25). The proportion of the N. gonorrhoeae NCCP11945 (GenBank accession number NC_011035.1) and C. trachomatis D/UW-3/CX (GenBank accession number NC_000117.1) reference genomes covered at ≥10-fold depth is reported as a summary of the proportion of the genome likely to have informative coverage given the inherent up to 10% base inaccuracy of ONT reads.

### Data availability.

Nanopore sequence read data generated from simulated and clinical infections are available from NCBI/EBI under study accession number PRJEB35173.

## RESULTS

### N. gonorrhoeae DNA extraction and human DNA depletion.

The four DNA extraction methods tested in simulated infections yielded various amounts of N. gonorrhoeae DNA. For example, in N. gonorrhoeae NAAT-negative urine samples spiked with 10^5^ CFU/ml of an N. gonorrhoeae reference strain, ethanol precipitation and the MagMAX kit yielded larger amounts of N. gonorrhoeae DNA; samples contained a median (interquartile range [IQR]) of 695 (60 to 2,020), 318 (68 to 595), 588 (16 to 1,037), and 180 (41 to 241) copies/ml of *porA* across all human DNA-depletion methods using ethanol precipitation, i-genomic, MagMAX, and QIAamp kits, respectively. Figure S2A in the supplemental material shows *porA* qPCR results for all spike concentrations tested. The amount of hands on time and total time taken for each of the four DNA extraction methods and their approximate cost are shown in Table S1 in the supplemental material.

Across all DNA extraction methods, the MolYsis Basic5 kit or saponin-based differential lysis successfully depleted the most human DNA. Comparing the amount of human DNA present without depletion to that present following depletion with these two methods, the median (IQR) percentage of human DNA remaining after depletion was 0.3% (<0.1% to 0.7%) and 1.1% (0.4% to 2.3%), respectively. Differential centrifugation did not lead to any observable depletion of human DNA and instead enriched for human DNA by 154% (108% to 277%) (Fig. S2B).

Saponin-based differential lysis produced the highest ratio of N. gonorrhoeae to human DNA across all the spikes and for all the DNA extraction protocols tested, with little observable difference between the four extraction methods (Fig. S2C). The QIAamp kit produced a higher ratio of N. gonorrhoeae to human DNA at the lowest spiked amount and the most consistent results overall. From these results and considering ease of sample processing within the laboratory with each of the protocols tested, we chose to use the saponin-based differential lysis and nuclease digestion followed by the QIAamp UCP pathogen mini kit as our laboratory method for subsequent experiments.

### Limit of detection.

**FIG 1:**
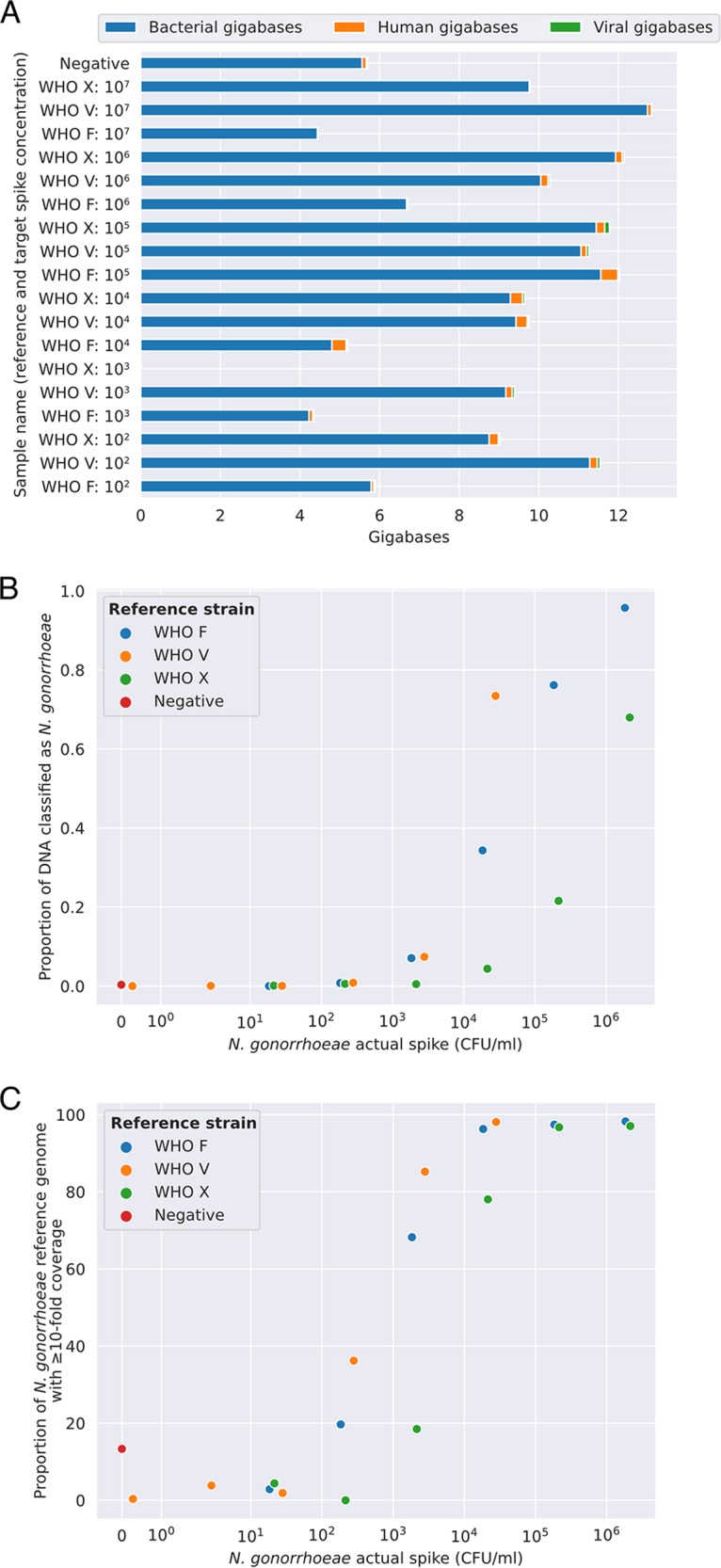
N. gonorrhoeae-simulated infections; limit of detection using Nanopore sequencing. (A) The proportion of sequenced reads classified as human, bacterial, or viral. (B) The proportion of bacterial bases classified as N. gonorrhoeae, and (C) the proportion of the NCCP11945 N. gonorrhoeae reference genome covered at ≥10-fold depth. For WHO V (orange markers), the actual spike concentration achieved was lower than the targeted concentration (see Fig. S3).

In these experiments, we initially targeted N. gonorrhoeae reference strain spikes ranging in concentration from 10^2^ to 10^7^ CFU/ml; however, the final concentrations achieved ranged from approximately 10^1^ to 10^6^ CFU/ml (see Fig. S3 in the supplemental material for comparison of target and actual spiking concentrations). Samples generated between 4 and 13 gigabases (Gb) of taxonomically classified sequence data (with the exception of one sample that failed library preparation and generated no sequence data). The majority of reads were classified as bacterial in all samples sequenced (Fig. 1A; see Fig. S4 in the supplemental material for a breakdown of the most common bacterial species identified). There was no relationship between the total number of bacterial reads and the concentration of the N. gonorrhoeae spike. This finding reflects the high concentrations of background bacteria present, such that at low spike concentrations the bacteria sequenced were predominantly other species, whereas at higher spikes N. gonorrhoeae sequence dominated (Fig. 1B). The proportion of reads classified as human was <5% for all samples and <1.5% for 10/18 samples. Bases classified as N. gonorrhoeae were detected in all samples spiked at ≥10^3^ CFU/ml (Fig. 1B). On mapping, we observed coverage at ≥10-fold depth of ≥75% of the NCCP11945 reference genome in all samples spiked with ≥10^4^ CFU/ml (Fig. 1C). Samples spiked at ≥10^5^ CFU/ml achieved a median (IQR) coverage breadth of 98% (97.2% to 98.1%) of the N. gonorrhoeae reference genome at an average coverage depth of 2,730 (1,133 to 4,027). We observed reads classified as N. gonorrhoeae in our unspiked negative-control urine sample, which mapped to <14% of the whole reference genome at a depth of ≥10-fold (see Fig. S5 in the supplemental material). However, no similar contamination was seen in six subsequent negative urine sample sequences (see Fig. S6B in the supplemental material).

### Detection of N. gonorrhoeae in the presence of Chlamydia trachomatis.

Our protocol also allowed direct detection of both N. gonorrhoeae and C. trachomatis DNA in simulated coinfections. DNA from chlamydia NAAT-positive urine spiked with N. gonorrhoeae isolates was extracted by using the QIAamp kit or mechanical lysis followed by ethanol precipitation, both following saponin treatment. Approximately 1.2 to 12.5 Gb of taxonomically classified sequence data were generated from 16/18 samples sequenced; two samples performed suboptimally in the library preparation with <400 megabases (Mb) generated. The majority of reads were classified as bacterial in all samples sequenced. The proportion of reads classified as human was slightly higher than with the C. trachomatis/N. gonorrhoeae NAAT-negative spiked urine, although it was <29% in all but one case (Fig. S6A). A total of ≥94% of the N. gonorrhoeae genome was covered at ≥10-fold in 3 of 4 samples spiked at ≥10^4^ CFU/ml, suggesting that although a greater proportion of the sequencing reads were classified as human, it is still possible to get both good breadth and depth of coverage of the N. gonorrhoeae genome in the presence of coinfection and inflammatory cells (Fig. S6B). Bases classified as C. trachomatis were observed for 12/18 of the chlamydia NAAT-positive spiked urine DNA extracts (derived from all 3 original urine samples) (Fig. S6C). However, sufficient sequence data to cover the majority of a C. trachomatis reference genome was only obtained in 5/18 extracts, which were all from the same original urine sample (Fig. S6D). Additional species, including Acinetobacter spp. and Streptococcus pseudopneumoniae, were identified in all three urine samples at high proportions (see Fig. S7 in the supplemental material), which likely hindered the recovery of C. trachomatis.

### Sequencing speed.

**FIG 2:**
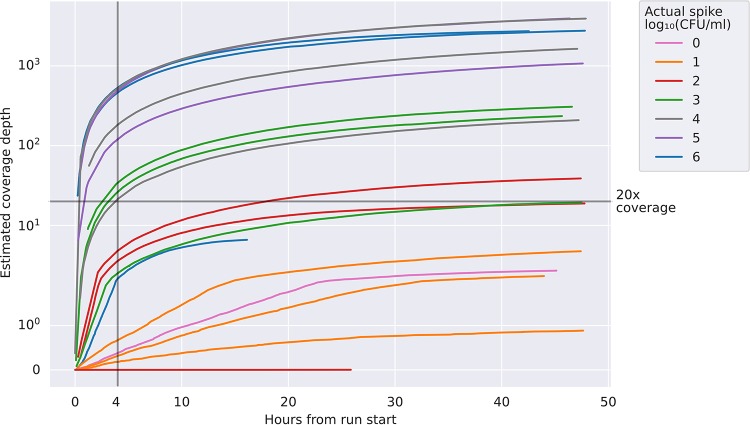
Sequencing speed in 18 N. gonorrhoeae and C. trachomatis NAAT-negative urine samples spiked with N. gonorrhoeae. The actual spiking concentration achieved is rounded to the nearest order of magnitude for the purposes of the legend. Estimated coverage depth is calculated as the number of bases of N. gonorrhoeae DNA sequenced divided by the length of the reference genome.

Figure 2 shows the sequencing time taken to achieve an estimated given coverage depth of the reference genome for the 18 N. gonorrhoeae and C. trachomatis NAAT-negative urine samples spiked with N. gonorrhoeae that were depicted in Fig. 1. For samples spiked with ≥10^4^ CFU/ml >20-fold coverage depth was typically achieved in ≤4 hours from starting sequencing.

### Performance in N. gonorrhoeae infection.

**FIG 3:**
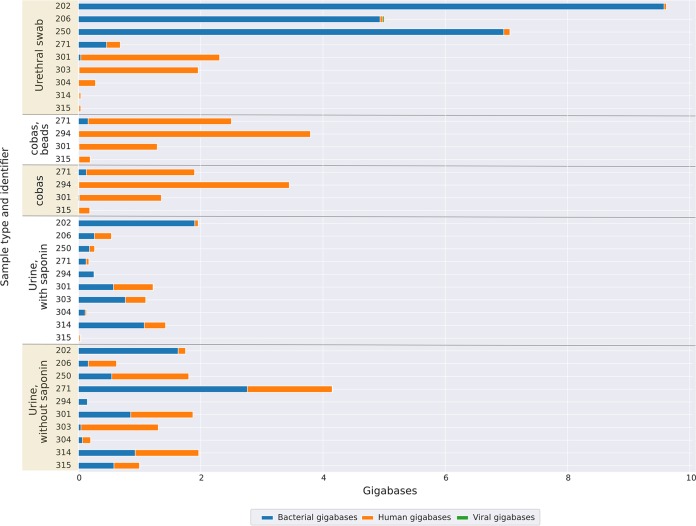
Performance in clinical samples positive for N. gonorrhoeae; yield of bacterial, human, and viral DNA sequenced. Results from 10 participants are shown, including where available reads obtained from a urethral swab, a urine sample collected into a cobas lysis buffer (processed with and without mechanical lysis with beads), and a urine sample collected in a universal container with a boric acid additive processed with and without treatment with saponin. The results present numbers of human reads and total reads, including data demultiplexed by porechop and guppy; all other analyses are based on guppy data alone (see Materials and Methods for details).

**FIG 4:**
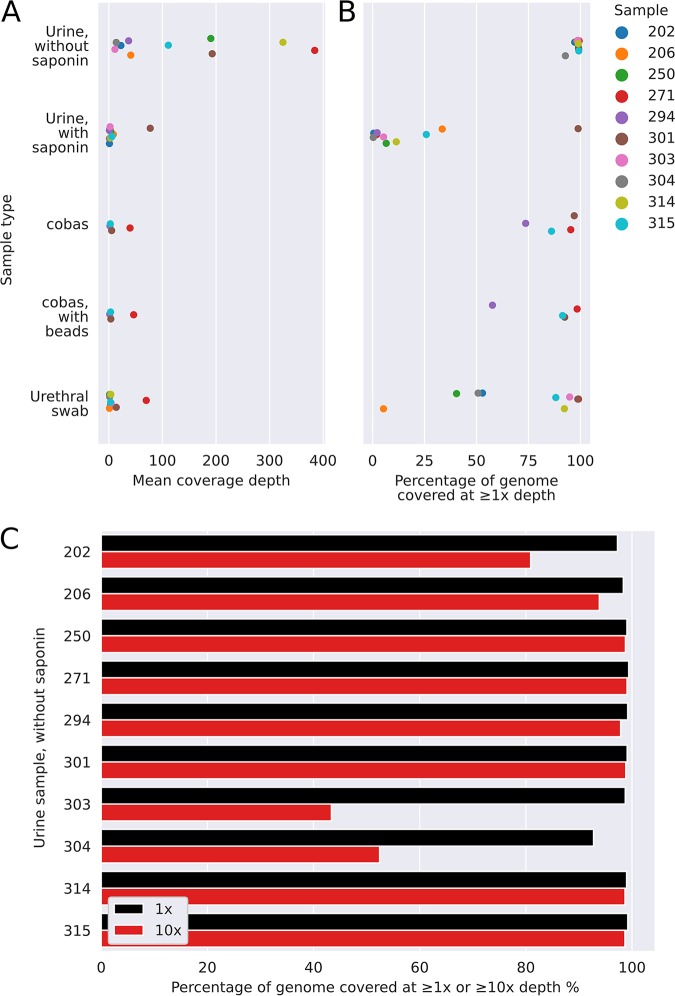
Performance in clinical samples positive for N. gonorrhoeae, including coverage breadth and depth. (A) The mean coverage depth achieved for samples processed by one the five methods tested, i.e., the total number of bases of sequence generated divided by the length of the NCCP11945 N. gonorrhoeae reference genome. (B) The proportion of the NCCP11945 N. gonorrhoeae reference genome covered by at least one read. (C) Data from urine samples processed without saponin treatment.

We extracted and sequenced DNA from 10 individual N. gonorrhoeae NAAT-positive urine samples using the QIAamp kit with and without prior human DNA depletion with saponin. All samples sequenced contained detectable N. gonorrhoeae DNA. In contrast to simulated infection, samples processed without saponin had higher yields of bacterial (Fig. 3) and N. gonorrhoeae DNA (Fig. 4). The length of the N. gonorrhoeae genome is approximately 2 Mb. Without saponin, the quantity of sequence data per sample classified as N. gonorrhoeae ranged from 26 Mb to 879 Mb, representing between 4% and 81% of total bacterial bases and corresponding to a median (IQR) [range] N. gonorrhoeae genome coverage breadth of 99.0% (98.5% to 99.2%) [92.8% to 99.4%] at a by-sample mean depth of 76 (26 to 192) [11 to 384] (Fig. 4). Median reported Phred-scaled base quality scores ranged from 20.2 to 22.7, i.e., 1 error per 105 to 186 bases sequenced (although these estimates are unlikely to be well calibrated) (26) (see Table S2 in the supplemental material for a listing of all quality metrics generated).

**FIG 5:**
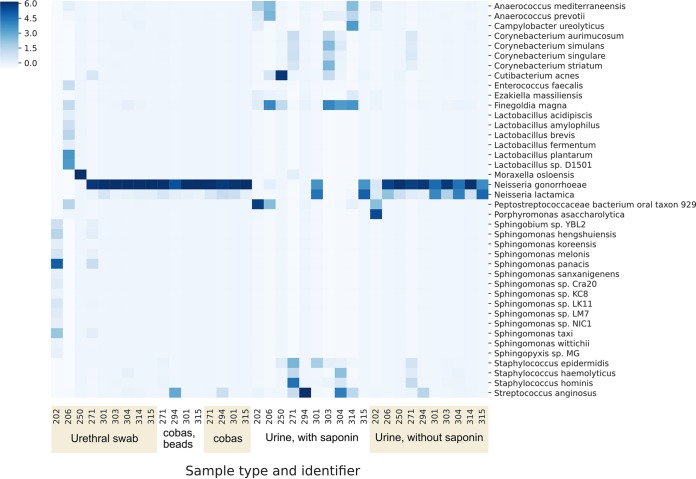
Performance in clinical samples positive for N. gonorrhoeae; relative proportions of species sequenced per sample. The Z-score, denoted by shade, for each taxon is the number of standard deviations above the mean number of bases per taxon for each sample.

At least 92.8% of the reference genome was covered by ≥1 read in all 10 samples and ≥98.4% of the reference genome in 8 samples (Fig. 4C). In 7 samples ≥93.8% of the reference genome was covered at a depth of ≥10-fold. We explored predictors of successful sequencing (see Table S3 in the supplemental material). Within the limits of the small study size, there was no relationship between the percentage of the reference genome with ≥10-fold coverage depth and NAAT threshold cycle (*C_T_*) values, the time between collection and processing, the concentration of DNA loaded on to the flow cell postamplification, or the number of active flow cell pores at the start of sequencing (all Spearman’s rank *P* > 0.15). In the 3 samples that did not achieve ≥93.8% coverage at 10-fold depth, 1 had high levels of contaminating bacteria DNA, namely, sample 202 (Porphyromonas asaccharolytica) (Fig. 5). In another, sample 303, the proportion of human reads was higher than in other samples (i.e., 97%) compared to a median (IQR) of 52% (30% to 69%). The final sample, 304, had a relatively poor sequence yield overall (0.2 Gb of data) compared to a per-barcode median (IQR) of 1.6 (0.6 to 2.0) Gb.

With saponin, the number of bases classified as N. gonorrhoeae ranged from 0.01 Mb to 177 Mb, representing <0.01% to 41% of total bacterial bases, with similar overall base quality scores to without saponin. The median (IQR) [range] N. gonorrhoeae genome coverage breadth was 5.9% (2.0% to 22.2%) [0.3% to 98.9%] at an average depth of 3 (1 to 5) [1 to 77]. Therefore, while saponin was effective a reducing the percentage of bases classified as human from a median from 52% to 25% (Fig. 3), this was not sufficient to offset the detrimental effect on N. gonorrhoeae in clinical samples.

Figure 5 shows the most commonly identified species across the five sample types (urethral swabs, cobas sample tubes with and without mechanical lysis with beads, and urine samples with and without saponin treatment). For 9 of the 10 urine samples processed without saponin treatment, N. gonorrhoeae was the most abundant species sequenced, with reads classified as Neisseria lactamica likely representing a taxonomic misclassification of reads from N. gonorrhoeae. Samples processed with saponin showed differential depletion of N. gonorrhoeae relative to other bacteria.

The large majority of bacterial reads were from N. gonorrhoeae in most urethral swab and cobas tube samples (Fig. 5). However, these samples contained smaller amounts of the total bacterial DNA sequence (Fig. 3), resulting in fewer sequenced N. gonorrhoeae bases and only a limited coverage of the reference genome (Fig. 4). By sequencing direct from cobas PCR medium tubes, without mechanical lysis with beads, the number of N. gonorrhoeae bases ranged from 3.7 Mb to 87 Mb, representing between 58% and 94% of total bacterial reads and resulting in genome coverage breadth from 74% to 97% at a per-sample mean depth from 2 to 40. Results with mechanical lysis were similar. Similarly, the number of bases classified as N. gonorrhoeae from urethral swabs ranged from 0.2 Mb to 162 Mb, representing between <0.01% and 91% of the total bacterial bases. The median (IQR) [range] genome coverage breadth was 88.2% (50.1% to 94.8%) [5.3 to 99.1] and depth 3 (1 to 4) [1 to 70].

### Coverage of chromosomal genes involved in antimicrobial resistance.

The median coverages of *penA*, *mtrR*, 23S (each of the four copies), *gyrA*, *gyrB*, *parC*, *ponA*, *pilQ*, and *rpsJ* are given for all clinical samples sequenced in Table S2. For the 10 urine samples processed without saponin treatment, ≥98% of each of the genes was sequenced to a depth of ≥1 read in all samples. The number of samples with ≥99% of the gene(s) with ≥10-fold coverage depth was 7 for all four 23S rRNA genes, 10 for *gyrA*, 9 for *penA*, and 6 for *mtrR*.

## DISCUSSION

We demonstrate that it is possible to extract and sequence sufficient quantities of N. gonorrhoeae DNA directly from urine samples from men with symptomatic urethral gonorrhea to achieve nearly complete reconstruction of the N. gonorrhoeae genome. It was possible to achieve coverage of ≥92.8% of the genome in all 10 patient samples and ≥93.8% coverage breath at ≥10-fold coverage in 7 (70%) samples. As we ran two experiments per flow cell for these clinical samples, potentially higher sequence yields could be achieved using a separate flow cell for each sample. Through simulated infections, we demonstrate that if N. gonorrhoeae is present at ≥10^4^ CFU/ml, sequencing of the large majority of its genome can be frequently achieved.

Our initial experiments with spiked N. gonorrhoeae NAAT-negative samples showed the four DNA extraction methods tested were broadly comparable and potentially any could be applied for sequencing direct from urine samples. However, we obtained contrasting results from attempted human DNA depletion in simulated and actual infections. In simulated infections, freshly cultured N. gonorrhoeae was spiked into urine samples, and DNA was extracted; in this setting, both saponin and the MolYsis kits improved N. gonorrhoeae DNA yields by differentially depleting human DNA, the former to a greater extent. However, in actual infections, N. gonorrhoeae appears to have been more susceptible to lysis by saponin, possibly reflecting damage to N. gonorrhoeae cells in storage, in transport, and by host inflammatory cells. This resulted in higher yields of N. gonorrhoeae DNA when saponin treatment was omitted.

The depth and breadth of coverage of the N. gonorrhoeae genome achieved in the majority of samples spiked with ≥10^4^ CFU/ml of N. gonorrhoeae and in N. gonorrhoeae NAAT-positive clinical samples should make the detection of antimicrobial resistance determinants possible, as well as comparisons of genomes for transmission tracking. We demonstrate similar levels of coverage of key chromosomal genes involved in antimicrobial resistance to coverage levels in the rest of the genome. In urine samples processed without saponin treatment, all chromosomal resistance genes analyzed were covered by ≥1 read over ≥98% of their length, and *penA*, 23S rRNA, and *gyrA* had ≥10-fold coverage over ≥99% of their length in ≥7 samples. It is also theoretically possible to identify plasmid-mediated resistance; however, we focused here on resistance determinants important for commonly used antibiotics, such as ceftriaxone, azithromycin, and ciprofloxacin, which are chromosomal.

The relatively high per base error rate of Nanopore sequencing means specific bioinformatic approaches are required to produce a consensus genome without an unacceptable number of false variants. Although reported error rates generated by the base-calling software used were approximately 1%, these are likely to be overly optimistic. A previous evaluation comparing Nanopore data to known sequences estimated the error rate at 6% (26). Where sequencing errors occur at random, this can be compensated for by achieving sufficient sequencing depth, e.g., ≥10-fold as achieved in the majority of our clinical samples, such that the true base at each position dominates. Follow-up work on robust identification of consensus sequences is an area of active research at present.

When initially planning the study, we considered that contamination with human DNA would be the principal technical challenge to be overcome. Although the clinical samples from N. gonorrhoeae infection tested still contained human DNA, in the majority of samples, sufficient N. gonorrhoeae DNA was present for successful sequencing without specific human DNA depletion. However, large amounts of human DNA present in one clinical sample resulted in reduced genome coverage. The presence of high levels of other bacterial species impaired the yield of pathogen DNA in simulated infections and in one of the samples from clinical N. gonorrhoeae infection. For simulated infections, this is likely because this study relied on samples discarded after routine testing. As such, these samples typically spent several days at ambient temperature in transport and in the laboratory, which allowed time for bacterial overgrowth to occur. This same issue was present in study participant samples, albeit to a lesser extent, as samples from Brighton were couriered overnight at ambient temperature to Oxford. The large amounts of DNA from other bacteria in one of the clinical samples occurred despite the use of boric acid as an additive to reduce growth. We also tested if collection of urine directly into an unselective cell lysis buffer (cobas PCR medium tubes) would prevent bacterial overgrowth. This approach was successful in preventing contamination with other bacterial DNA (Fig. 5); however, it also better preserved human DNA (Fig. 3) such that the total amount of bacterial DNA sequenced (Fig. 3) and, hence, yield of N. gonorrhoeae DNA (Fig. 4A and B) was lower using this approach. As we only had access to the cobas PCR medium tubes and not the complete cobas platform, we could not test the performance of sequencing following DNA extraction by the platform. N. gonorrhoeae DNA yields from urethral swabs were also low, which may represent low numbers of organisms collected, particularly as obtaining these swabs required a second urethral swab (in addition to that taken for routine culture), which was potentially uncomfortable for participants. We were only able to assess the performance of our approach in a limited number of simulated N. gonorrhoeae/C. trachomatis coinfections; further work is needed in the future to assess its performance in coinfected patients.

Although our results provide a proof of principle, the applicability of sequencing in its current form is also limited by the time taken to prepare samples for sequencing; this requires up to 10 hours, largely due to the need for prolonged PCR amplification of very low quantities of input DNA. Current reagent costs are also >$400 per sample; while costs could be reduced by multiplexing multiple samples per flow cell, it would reduce sensitivity. Additionally, established bioinformatic workflows will be required to enable future applications of sequencing as a clinical diagnostic; they will need to be sufficiently accessible and robust to be used by those without specialist bioinformatic expertise.

Our results also highlight another current limitation of metagenomic sequencing, which is the potential for contamination, particularly as the approach relies on nonselective amplification of all DNA present. In one of our seven negative-control urine samples, around 15% of the N. gonorrhoeae reference genome was covered at high depth; however, the coverage was very uneven (Fig. S5). This partial coverage of the reference genome may have arisen from contamination with PCR amplicons. This reinforces the need for metagenomic sequencing-based studies to include appropriate negative controls. Additionally, confirmation of the presence of N. gonorrhoeae may also require achieving coverage of a substantial proportion of the reference genome, and further metagenomic sequencing studies of patients with and without N. gonorrhoeae infection are required to assess this and determine thresholds for robustly identifying infection. Larger parallel studies using culture-based diagnostics alongside sequencing will also be required to determine the accuracy of antimicrobial resistance detection.

The focus of the manuscript was to optimize laboratory methods, which we have successfully achieved. This work provides a firm foundation for developing bioinformatic methods for confirming the presence of N. gonorrhoeae and resistance gene characterization using Nanopore data. If this can be achieved, same-day metagenomic diagnosis of gonorrhea infection and antimicrobial resistance is likely to be possible.

## Supplementary Material

Supplemental file 1

Supplemental file 2
