# Supplementary material for: Optimizing DNA Extraction Methods for Nanopore Sequencing of Neisseria gonorrhoeae Directly from Urine Samples
Source: J Clin Microbiol. 2020 Feb 24;58(3):e01822-19. doi: 10.1128/JCM.01822-19 (PMC7041563; doi:10.1128/JCM.01822-19)
Supplement: Supplemental file 1 [file JCM.01822-19-s0001.pdf]

Optimizing DNA extraction methods for Nanopore sequencing of *Neisseria gonorrhoeae* direct from urine samples: Supplemental material

Supplementary Table

Table S1. Summary of hands on time and total time for each of the four human DNA depletion methods, DNA extraction methods and ONT sequencing preparation, and their approximate cost per sample. Total time includes all incubation steps longer than 15 minutes. Following the times shown ONT sequencing was run for up to 48 hours. Initial results were typically available faster (Figure 2). Costs are based on actual reagent prices paid during the study.

Method	Hands on time (single sample)	Hands on time (per batch)	Total time (per batch)	Cost per sample (US $)
**Human DNA depletion**				
MolYsis Basic5	45 min	1hr 40 min	1hr 40 min	10.40
Saponin + nuclease	1hr 10min	2 hr	2 hr	4.55
Differential centrifugation	30 min	1 hr 10 min	1 hr 10 min	0.13
No treatment	25 min	35 min	35 min	0.13
**DNA extraction**				
MagMAX Total Nucleic Acid Isolation Kit	1hr 15 min	3hs 25 min	3hr 25 min	3.51
Mechanical lysis + ethanol Precipitation	1hr 40 min	2hr 40 min	4hr	4.55
QIAamp UCP Pathogen Mini Kit	1hr 30 min	2hr 50 min	2hr 50 min	8.06
i-genomic Urine DNA Extraction Mini kit	45 min	1hr 50 min	1hr 50 min	5.20
**Sequencing preparation and flowcell cost**				
ONT Rapid PCR barcoding kit + flowcell	1hr 10 min	1hr 40 min	5hr 25 min	409.50
**Saponin + QIAamp laboratory method**	3hr 50 min	6hr 30min	10hr 15 min	422.11

Table S2. Quality metrics for clinical sample sequences. Please see the separate excel spreadsheet provided. The number of human bases and reads were determined from porechop demultiplexed data (see methods), all other metrics were determined from guppy demultiplexed data.

Table S3. Potential predictors of successful sequencing for 10 urine samples. Table uses pooled reads from samples processed with and without saponin. Following transport overnight to the laboratory at ambient temperate, samples were stored at 4°C between collection and DNA extraction.

Sample	NAAT CT value, opa	NAAT CT value, pilin	Days between sample collection and DNA extraction	DNA loaded on to flowcell, fmol	Nanopore initial QC pores	Percentage of reference genome with ≥1x depth coverage	Percentage of reference genome with ≥10x depth coverage
**202**	20.3	22.1	44	50	1170	97.2%	80.9%
**206**	22.7	22.6	45	50	1250	98.4%	93.8%
**250**	21.0	22.0	24	50	1337	99.0%	98.8%
**271**	28.8	28.7	6	60	1474	99.4%	99.1%
**294**	20.5	20.5	6	60	1613	99.2%	97.8%
**301**	22.3	22.6	27	50	1170	99.1%	98.8%
**303**	25.9	25.6	19	50	1402	98.7%	43.4%
**304**	21.5	23.1	39	41	1171	92.8%	52.4%
**314**	20.2	21.6	32	50	1587	99.0%	98.7%
**315**	18.8	20.1	25	50	1445	99.2%	98.7%

Supplementary Figures

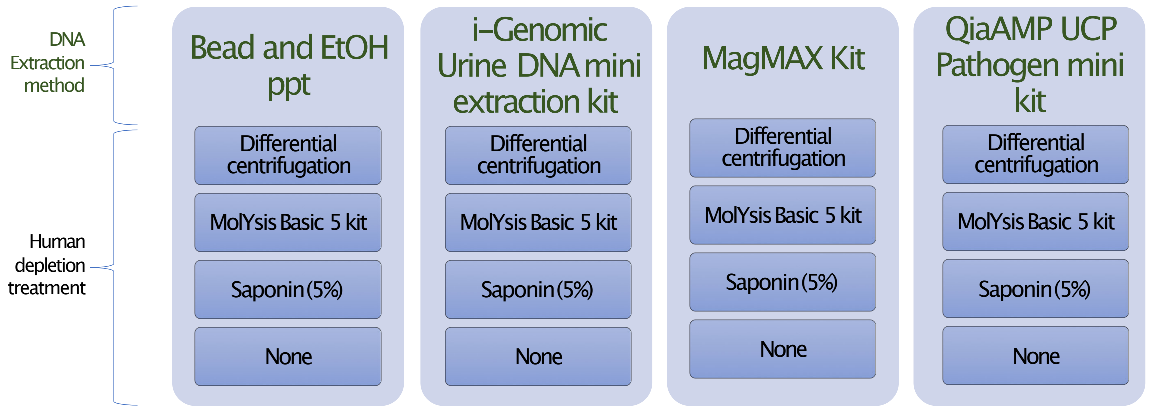

Figure S1. *N. gonorrhoeae* spiking experiments. *N. gonorrhoeae* NAAT-negative urine was pooled and spiked with approximately 10^3, 10^5 or 10^7 CFU/ml of reference strains WHO F, WHO V or WHO X. Each human DNA depletion method was tested in triplicate, at each spiked amount, with each DNA extraction method. A single, no-spike control was included per depletion method (160 extractions in total).

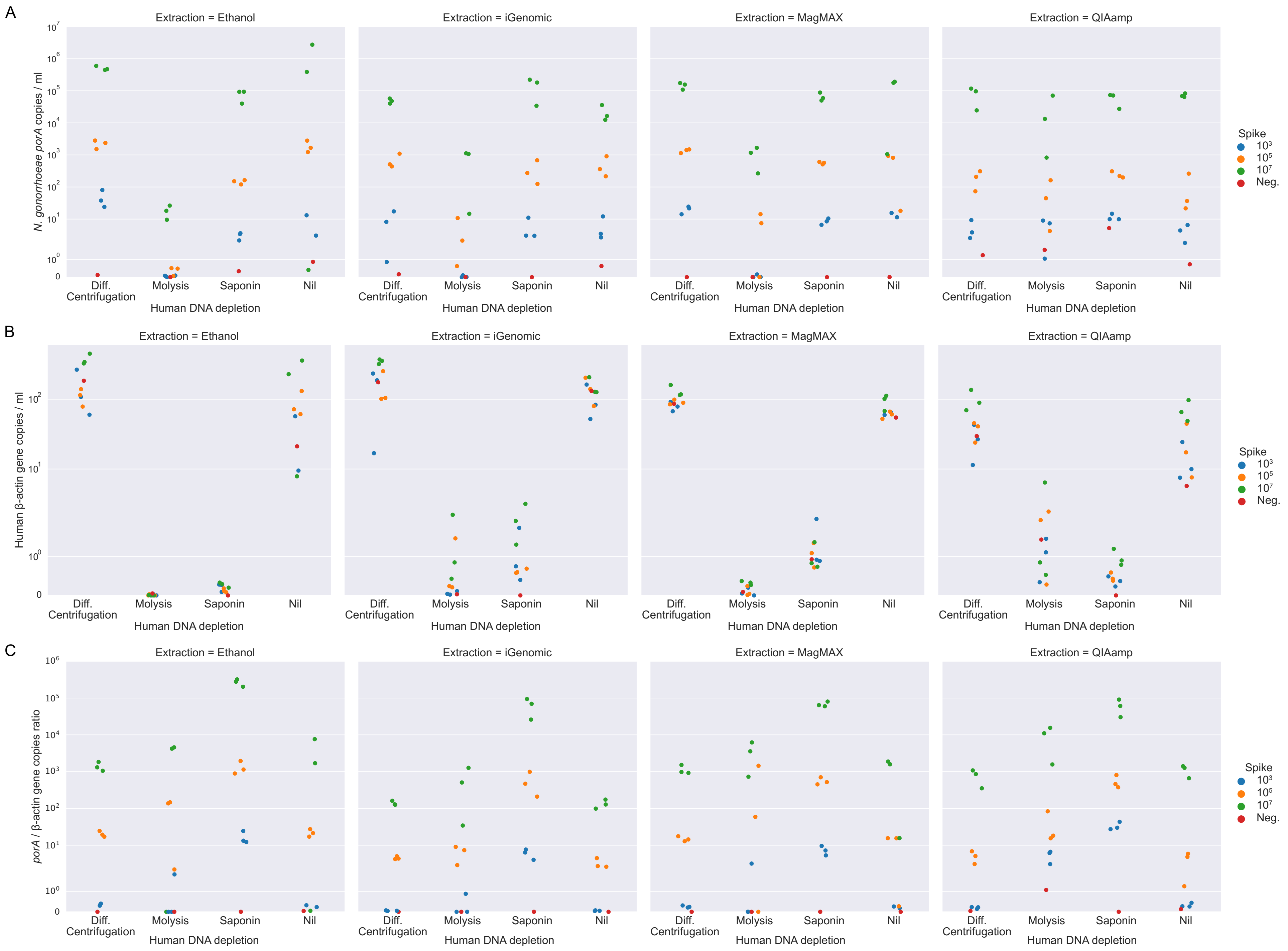

A
Extraction = Ethanol
Extraction = iGenomic
Extraction = MagMAX
Extraction = QIAamp
N. gonorrhoeae porA copies / ml
Diff. Centrifugation
Molysis
Saponin
Nil
Human DNA depletion
Spike
10^3
10^5
10^7
Neg.
B
Human β-actin gene copies / ml
C
porA / β-actin gene copies ratio

Figure S2. Performance of human DNA depletion methods and DNA extraction methods using qPCR. Row A shows the copies per ml of *N. gonorrhoeae porA*, row B shows the number of human β-actin copies per ml and row C shows the average proportion of *porA* to β-actin copies. Within each row, each of the 4 panels show different DNA extraction methods, and within each panel different human DNA depletion strategies are shown. Each point plotted is coloured by the target *N. gonorrhoeae* spike concentration, with each spike concentration tested in triplicate for each DNA extraction and human DNA depletion combination. Abbreviations for human depletion methods as follows: Diff. Centrifugation, differential centrifugation; MolYsis, MolYsis Basic5 kit; Saponin, saponin-based differential lysis; None, no human depletion. Data are not shown for the 10^3 CFU/ml WHO F spikes with no human DNA depletion, extracted with both ethanol precipitation and the MagMAX kit, due to a technical failure in the laboratory. Up to 8 copies of *porA* were seen in the negative samples extracted using the QIAamp kit, this probable low-level contamination is unlikely to have significantly impacted the spiked results for this kit.

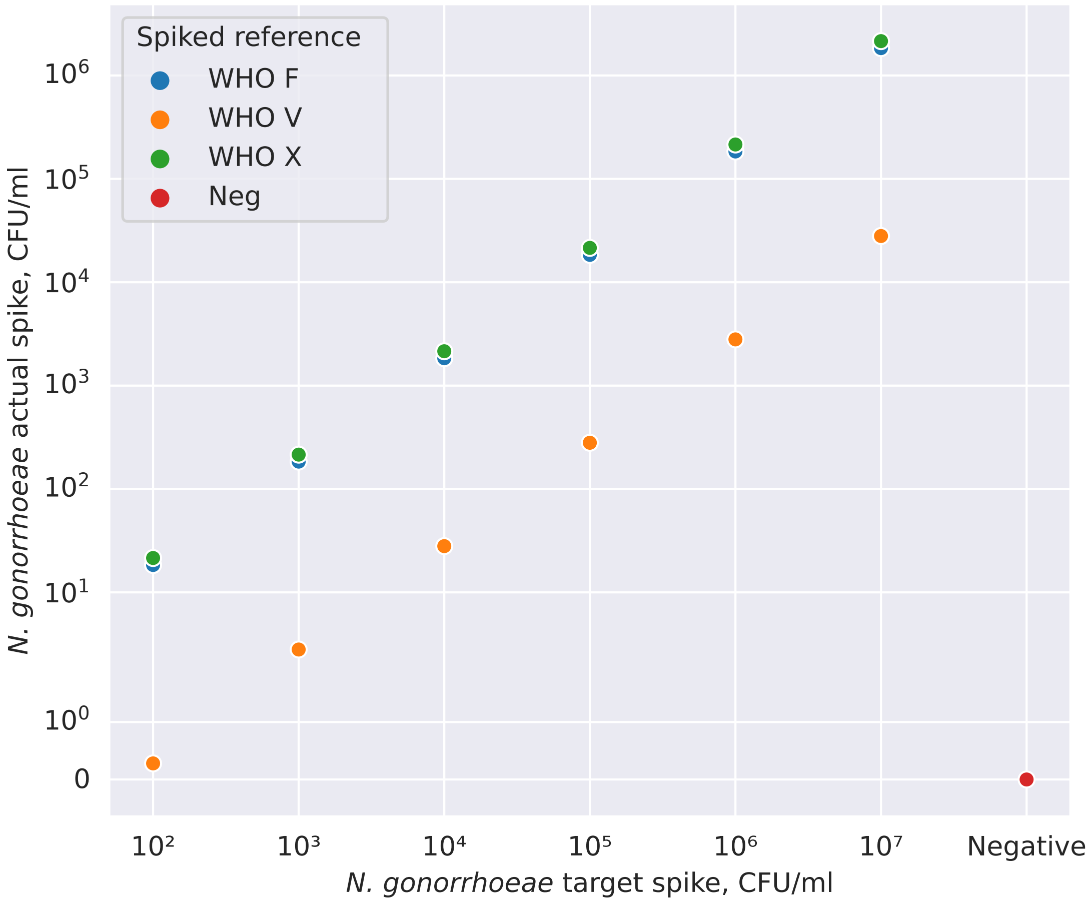

Figure S3. Target versus actual *N. gonorrhoeae* spiking concentrations. CFU/ml concentrations of the *N. gonorrhoeae* reference strains used in the limit of detection, in-depth spiking experiments.

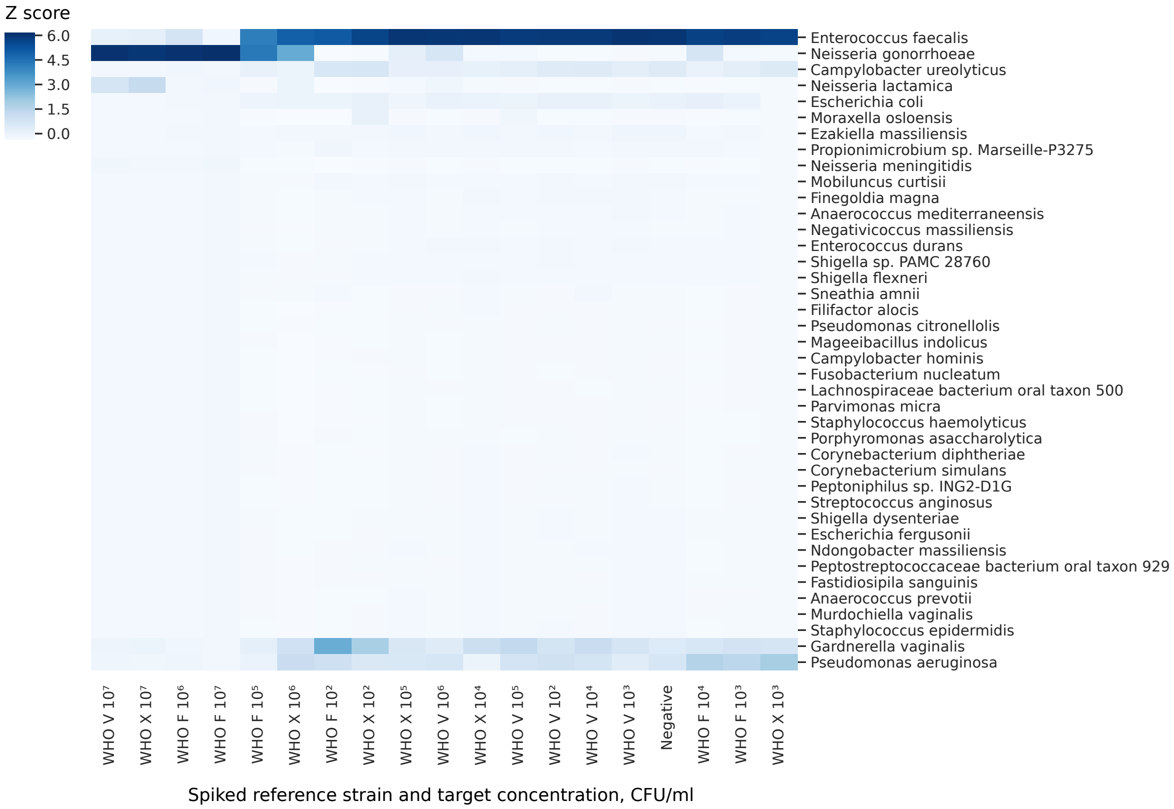

Figure S4. Common bacterial species sequenced in limit of detection, in-depth spiking experiments. The x-axis shows the spiked reference strain and concentration in CFU/ml. The z-score is the number of standard deviations above the mean number of bases per taxon for the taxa shown on the y-axis.

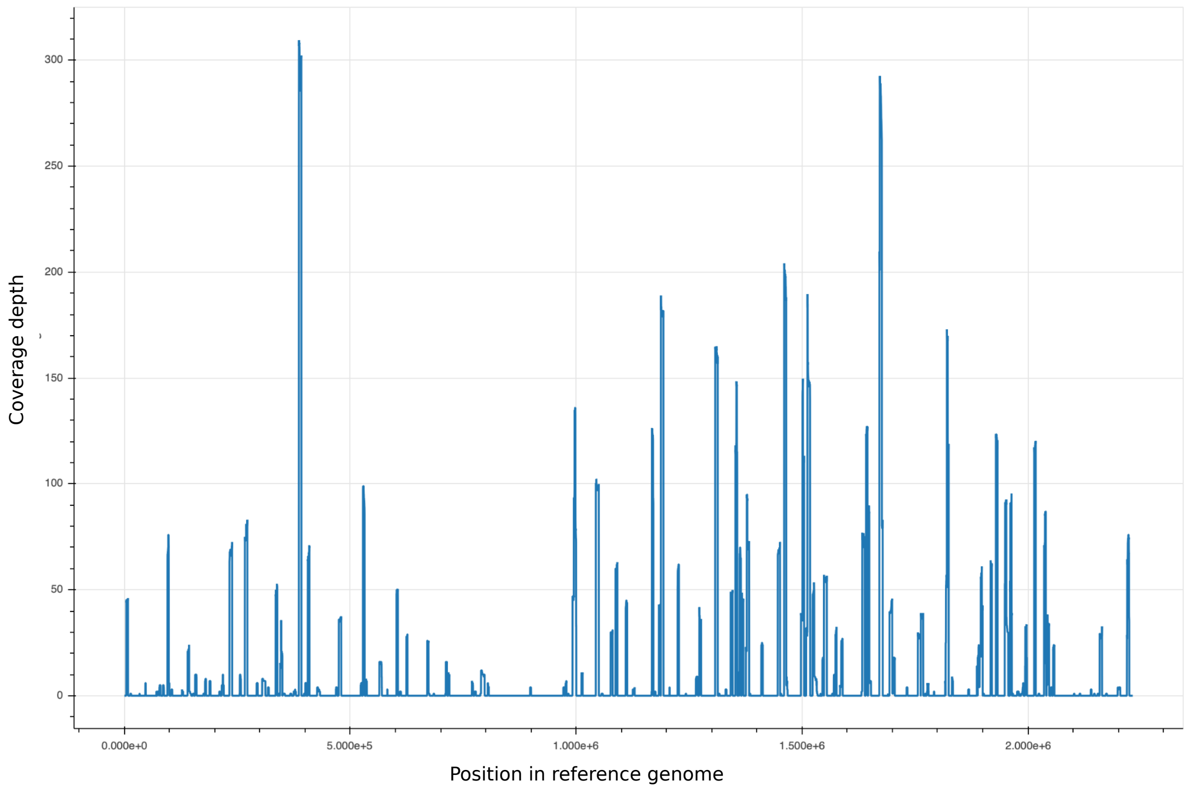

Figure S5. *N. gonorrhoeae* reference genome coverage observed in a negative control sample. Reads mapped to less than 24% of the reference genome at a coverage depth of 1-fold, and less than 14% at a coverage depth of ≥10-fold.

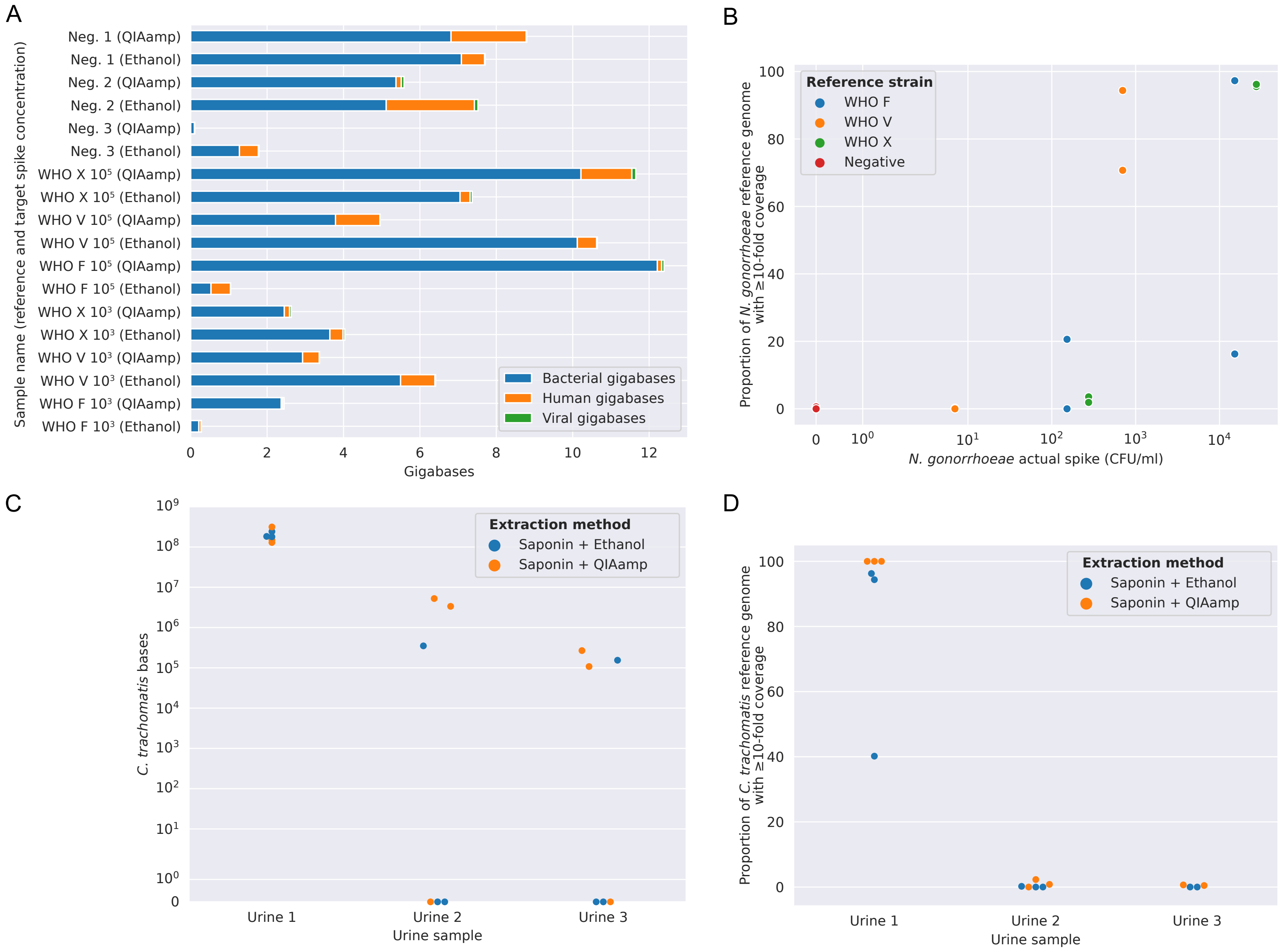

Figure S6. *N. gonorrhoeae* and *C. trachomatis* detection in spiked Chlamydia NAAT-positive samples. Chlamydia NAAT-positive urine collected from three patients was used. Target spikes of 10^2 and 10^4 CFU/ml of *N. gonorrhoeae* WHO F were individually spiked into 3 ml of urine from each patient. This was repeated for *N. gonorrhoeae* WHO X and WHO V and an un-spiked sample from each patient was used as a negative control (n=18). Panel A shows the proportion of sequenced reads classified as human, bacterial or viral. Panel B shows the proportion of the NCCP11945 *N. gonorrhoeae*

reference genome covered at ≥10-fold depth. Panel C shows the total number of *C. trachomatis* bases sequenced and panel D the proportion of the D/UW-3/CX *C. trachomatis* reference genome covered at ≥10-fold depth.

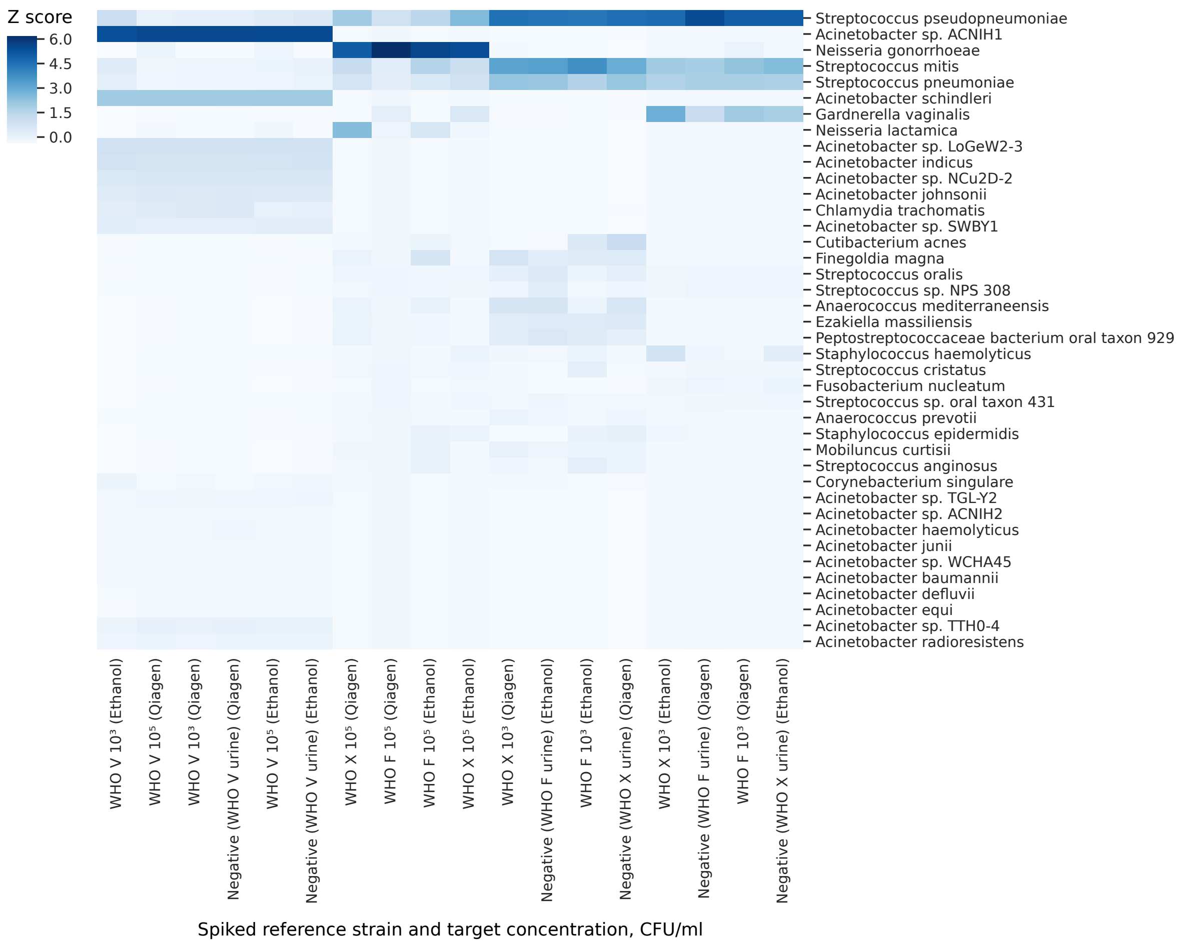

Figure S7. Detection of additional species in Chlamydia NAAT-positive samples. Cluster map of the top 40 additional species identified by sequencing in the three spiked Chlamydia-positive urines. The z-score is the number of standard deviations above the mean number of bases per taxon for the taxa shown on the y-axis.
